# Renal Stone and Its Diagnosis

**Published:** 1888-01

**Authors:** 


### RENAL STONE AND ITS DIAGNOSIS.

Since the operation of nephrectomy was first established by Simon, 1869, there have been, according to Gross, two hundred and thirty-three cases reported, with a mortality of 44.63 per cent. The records of the later work of surgeons in this line no doubt give better results than are indicated by a consideration of all cases, including those operated upon when the technique was less perfectly understood.

An operation apparently much more brilliant in results than nephrectomy is that of nephro-lithotomy, first performed by Mr. Henry Morris in 1880, for removal of a renal calculus. This has now been done over twenty times, with a mortality of only eight or ten per cent.

It seems, therefore, as if, by the help of modern renal surgery, kidney-stone could now be treated with satisfactory results. In a recent number of the *Practitioner*, Mr. Jordan Lloyd, of Birmingham, calls attention to some views of his own regarding this matter. One trouble, he thinks, in the surgical treatment of renal lithiasis, is that the diagnosis is quite as often made incorrectly as otherwise. In twenty-five instances kidneys have been "explored" for supposed renal calculus without any stone being found. The operators in three cases have not been deterred by the trivial fact of a false diagnosis, but have taken out the kidneys just the same. The kidney, like the ovary, has to pay its tribute to the mad frenzy of the modern record-making surgeon.

Mr. Lloyd thinks that fewer mistakes will be made, first, after the knowledge of clinical symptoms is more complete, and, second, when present misconceptions of the anatomy of the renal pelvis are corrected.

As to the latter point, he claims to have demonstrated, by casts taken from human kidneys, that the pelvis is an utterly different arrangement to what the text-books would lead us to believe; and that, instead of its being a "funnel-shaped membranous sac," chiefly located in the organ's interior, it really consists of a cluster of branching tubes; so that the procedure of exploring the kidney's interior, by means of a finger introduced into the pelvis through an opening in the kidney-substance, is practicable only in dilated kidneys, and is absolutely impossible when we are examining the interior of the viscus in anything approaching a normal condition. Mr. Lloyd has not yet found a healthy human kidney into the primary pelvic tubes of which he could introduce his index-finger. The precise anatomical arrangement of the ureter and pelvis is stated to be as follows: "The ureter as it approaches the kidney enlarges from its normal caliber—about that of a No. 10 English catheter—until it measures from one-third to half an inch in diameter, and immediately on entering the hilum it breaks up into two, or sometimes three, primary tubular branches, varying in diameter from a No. 10 to a No. 20 English catheter, and measuring from a half to one and a half inch in length. These in turn give off secondary tubes, smaller in size—some less than a line in width—which end in cup-shaped calyces. Sometimes tertiary branches, still smaller in caliber, shoot off from these secondary tubes to end in calyces. With such a conception of the structure of the kidney's interior, many of the occasional difficulties with regard to symptoms, prognosis, and treatment of renal calculus are made more easy to understand.

Mr. Lloyd contributes a method of reducing the number of "missed calculi." It consists essentially in cutting down on the kidney by a lumbar incision. A long tenotome is then thrust through the kidney into the lowest calyx. Through this opening is passed a child's bladder-sound, and the pelvis thoroughly explored.

The leading symptoms of renal stone, as given by the author in question, are as follows:

"Pain of a dull, heavy, dragging character, constantly located in one loin, sometimes shooting down the course of the ureter and along the spermatic cord to the testis, and always increased by exertion, is fairly diagnostic of stone; but, unfortunately for our art, the symptoms of disease are rarely true to type, their manifestations are capricious, and their inconsistencies are manifold.

"Pain may be present in both loins, and it then becomes very perplexing; or it may be referred to other and distant parts, so that the lumbar trouble may be altogether uncomplained of.

"It may be brought on by an injury. Much importance is attached to percussion of the region over the kidneys. The patient should stand upright, and the blow should be a sharp one. When a calculus is present the patient will complain of a stabbing pain, caused by the blow."—*The Medical Record.*

